# The Risk of Ischemic and Hemorrhagic Stroke in Head and Neck Cancer: A Longitudinal Cohort Study

**DOI:** 10.3390/cancers15133503

**Published:** 2023-07-05

**Authors:** Chulho Kim, Hyunjae Yu, Dong-Kyu Kim

**Affiliations:** 1 Department of Neurology, Chuncheon Sacred Heart Hospital, Hallym University College of Medicine, Chuncheon 24253, Republic of Korea; gumdol52@hallym.or.kr; 2 Institute of New Frontier Research, Division of Big Data and Artificial Intelligence, Hallym University College of Medicine, Chuncheon 24252, Republic of Korea; yunow3618@hallym.or.kr; 3 Department of Otorhinolaryngology-Head and Neck Surgery, Chuncheon Sacred Heart Hospital, Hallym University College of Medicine, Chuncheon 24253, Republic of Korea

**Keywords:** cancer, head and neck, stroke, cohort studies, incidence, risk

## Abstract

**Simple Summary:**

Several previous studies have shown that head and neck cancer may be associated with an increased risk of ischemic stroke but not hemorrhagic stroke. However, whether head and neck cancer causes an increased risk of developing ischemic or hemorrhagic strokes remains unclear. We found that patients with head and neck cancer had a significantly increased risk of overall and ischemic stroke development, although there was no significant difference in the incidence and risk ratio of hemorrhagic stroke. However, in the subgroup analysis, patients with oral cavity cancer had a significantly increased risk of both ischemic and hemorrhagic strokes compared to the general population. These findings suggest that clinicians should carefully monitor the development of stroke when they follow head and neck cancer survivors.

**Abstract:**

Several studies have shown that head and neck cancer is associated with an increased risk of stroke incidence. However, investigations of the association between the two diseases based on a matching cohort dataset are still limited. Therefore, we identified the incidence and risk of stroke in patients with head and neck cancer using a nationwide population-based cohort dataset. A total of 5248 individuals without cancer and 1312 with cancer were enrolled from the dataset after a 4:1 propensity score matching. We found that the incidence of overall stroke (per 1000 person years) was 19.29 for those with head and neck cancer, consisting of 17.32 as ischemic type and 2.74 as hemorrhagic type. Additionally, patients with head and neck cancer had a significantly increased risk of overall and ischemic stroke development but not hemorrhagic stroke. Moreover, the risk of stroke development over time was relatively high within the first year after the diagnosis of head and neck cancer. However, in the subgroup analysis, oral cavity cancer survivors showed an increased risk of both ischemic and hemorrhagic strokes. Therefore, our nationwide population-based cohort study suggests that clinicians should closely monitor patients with head and neck cancers for the early detection of stroke.

## 1. Introduction

Cancer-related stroke is one of the most important complications in patients with cancer. Previously, various studies have demonstrated that different cancer types have been shown to be associated with stroke [1,2,3]. As per a recent investigation based on meta-analysis although the risk of stroke decreases over time, the first year after the cancer detection represents the highest risk of the subsequent development of stroke [4]. To date, various studies have tried to investigate the cancer type that demonstrates a close relationship with the subsequent development of stroke subtypes, such as ischemic and hemorrhagic stroke. Previous studies have demonstrated that lung, breast, prostate, pancreatic, and urogenital cancer types showed an increased risk related to the ischemic type, whereas melanoma, renal cell carcinoma, choriocarcinoma, and colorectal cancer are strongly associated with a higher incidence of hemorrhagic stroke [1,5,6,7].

Generally, head and neck cancer is defined as the development of malignancy on the mucosal surfaces of the head and neck area, such as the sinonasal tract, nasopharynx, oral cavity, oropharynx, hypopharynx, and larynx. Epidemiological evidence has described that head and neck cancer accounts for approximately 3–5% of all cancer types in the United States [8,9]. In terms of treatment, surgery or radiotherapy alone is performed on patients who have localized head and neck cancer, but if patients have a more advanced form, physicians usually prefer the multimodal approach. For these reasons, several studies have attempted to elucidate the relationship between these two diseases. Previous studies have described that patients who underwent radiotherapy alone or in combination with other treatments showed a strong relationship with incident ischemic stroke events, but we could not find any studies regarding the increased risk of hemorrhagic stroke related to the radiotherapy [10,11,12,13,14,15,16].

Therefore, we tried to thoroughly evaluate the increased risk of ischemic and hemorrhagic stroke development in patients with head and neck cancer using well-matched samples extracted from a nationwide database. Since this dataset includes all types of medical conditions, we could determine the possible association between two specific diseases; we also controlled the suspected confounding factors, such as clinical status and demographic conditions.

## 2. Materials and Methods

### 2.1. Study Dataset

The Institutional Review Board of Hallym Medical University, Chuncheon Sacred Hospital approved the present study (IRB No. 2021-08-006) with waived written informed consent because this study used the NHIS dataset which consists of de-identified information. All authors adhered to the tenets of the Declaration of Helsinki. All authors confirmed that all types of data regarding the new findings of this study could be found in the present study’s content. Korea’s national health care system is a single payment system. Therefore, NHIS has records related to all Koreans’ medical practices. In addition, all insured persons in Korea pay insurance premiums in proportion to their household income, and all users are obliged to subscribe to the NHIS. In addition, the National Health Insurance Corporation issues a unique identification number at birth to identify individuals, which means that NHIS data cannot be omitted or duplicated. For this reason, the NHIS-NSC dataset can be said to be a standard cohort that reflects all Koreans. It is based on a 2.2% representative sample of Koreans and all sampling datasets consist of systematically stratified random samples with proportional assignment within each stratum. Additionally, previous studies performed a validation analysis on the cohort profile and showed a similar prevalence of major diseases every year [17,18]. Moreover, this cohort dataset has been widely used to elucidate the association between two certain diseases [19,20,21,22]. This indicates that the cohort dataset is highly reliable.

### 2.2. Study Design

**Figure 1:**
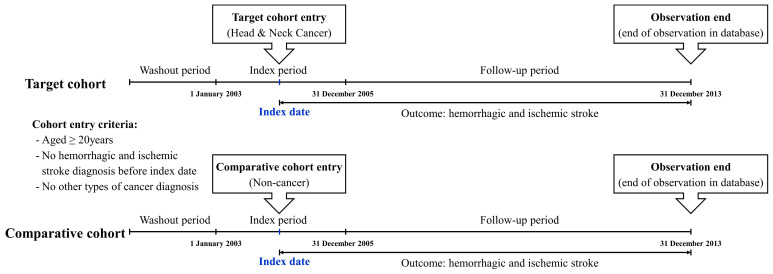
Description of the design of this retrospective cohort study.

**Figure 2:**
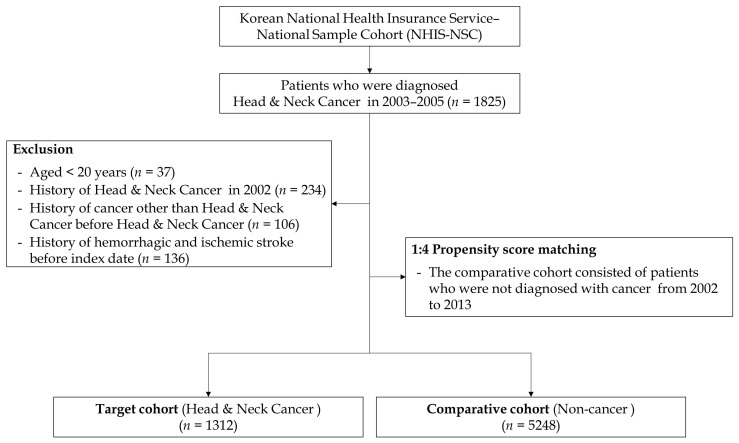
Flow chart of study enrollment.

We simply described the design of this retrospective cohort study (Figure 1). As we have shown, this study had a washout period of 1 year (January to December 2002) to remove the risk of stroke events before the participants’ enrollment. Next, to enroll participants of patients and comparison groups, we set the index period between January 2003 and December 2005. At this point, we defined the patients’ group as those who had diagnostic codes with C00–C14 and C30–C32 during the index period. We also excluded patients aged <20 years and those diagnosed with any type of stroke before head and neck cancer events during the index period. In addition, to elucidate the pure influence of head and neck cancer, we excluded individuals diagnosed with any other type of cancer. During the follow-up period, the incident stroke events (I60-63) or all-cause mortality were defined as the primary events for the end-point. If the patient had no events (meaning that the patient was still alive on the final follow-up period of the database), we censored those at this time point. To construct the matched comparison group (non-cancer), we randomly selected participants using the propensity score-matching technique as four those by one cancer patients. These patients were matched with the cancer group participants for individual variables and year of enrollment (cancer diagnosis). The flow chart of the participants’ enrollment process for each group is shown in Figure 2.

In this study, we have planned to select several independent variables, which are highly suspected confounding factors. Thus, we identified variables for age, sex, residence, household income, and comorbidities per each participant. Additionally, in the study based on claim data, the Charlson Comorbidity Index (CCI) was widely used to adjust the comorbidities; thus, we used the CCI to adjust comorbidities between the cancer group and the non-cancer group in this study.

### 2.3. Statistical Analysis

We defined incidence as the rate of new cases or events over a specified period for the population at risk for the event and calculated the incidence rates using the years-at-risk data of 1000 persons. In the present study, we calculated the person year differently according to the following three cases. First, in the case of death, the number of years from the date of first cancer diagnosis to the date of death. Second, in the case of a specific event, the number of years from the first diagnosis of cancer to the date of the first diagnosis of the specific event. Finally, in the case of no event, the number of years of cancer from the date of initial diagnosis to the end of the study. Additionally, Cox proportional hazard regression analyses were conducted to calculate the risk of following primary events. We presented the risk ratio as the hazard ratio (HR) and 95% confidence interval (CI). We also considered significance when the *p*-value was less than 0.05.

## 3. Results

### 3.1. Matching/Descriptive Portion of the Analysis

**Figure 3:**
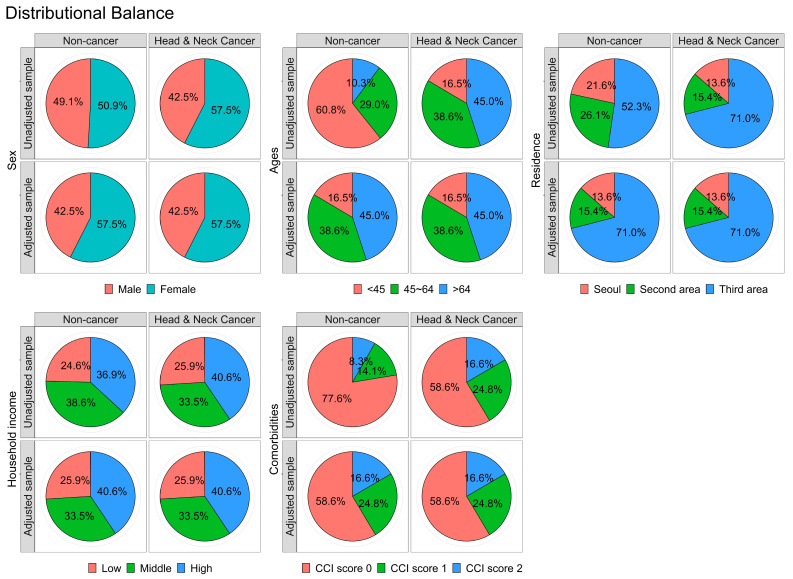
Confirmation of propensity scoring matching using the balance plot for all independent variables.

The head and neck cancer and non-cancer groups showed similar distributions with respect to all the covariates used for sample matching. In addition, we found no significant differences in any independent variables between the two cohorts (Table 1). Additionally, we performed a balance plot test to confirm appropriate matching (Figure 3). Based on these data, we conclude that cohort group matching in this study was performed appropriately.

### 3.2. Incidence Analysis

To investigate the incidence rate, we examined 44,523.2 person years in the non-cancer group and 9589.1 person years in the cancer group. The incidence of stroke in the cancer group was 19.29 per 1000 person years, whereas it was 13.41 per 1000 person years in the non-cancer group. Additionally, our findings revealed that the incidence rate in the cancer group was 2.74 for ischemic stroke and 17.32 for hemorrhagic stroke, respectively. We also found the incidence rate in the non-cancer group to be 12.13 for ischemic stroke and 1.86 for hemorrhagic stroke.

### 3.3. Risk Analysis

**Figure 4:**
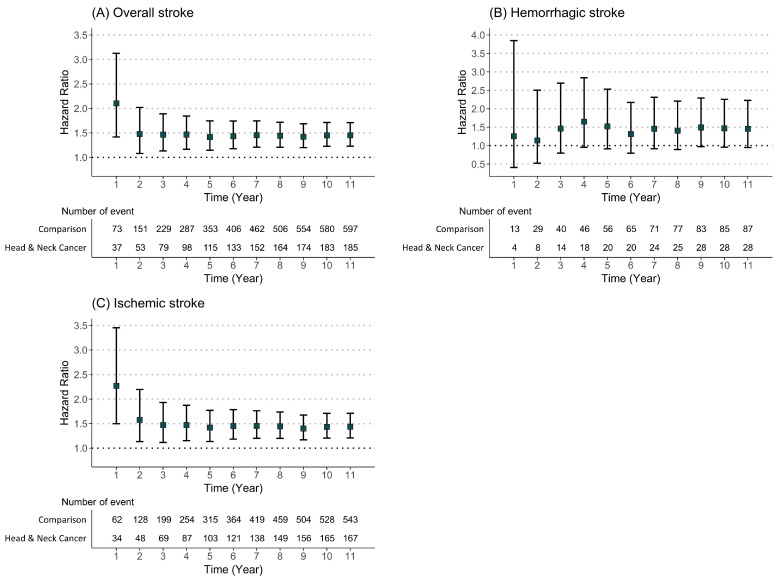
The risk of specific diseases in patients with head and neck cancer according to the follow-up period: (**A**) overall stroke; (**B**) hemorrhagic stroke; (**C**) ischemic stroke.

We also analyzed the HR for incident stroke events using Cox regression models during the 10-year follow-up period (Table 2). Our Cox analysis demonstrated a significant association between head and neck cancer and the development of stroke events (adjusted HR = 1.45, 95% CI = 1.23–1.71). In addition, the risk of ischemic stroke events in patients with head and neck cancer was 1.44 (95% CI = 1.21–1.71); however, we could not detect significant HR for hemorrhagic stroke in those. Moreover, we found that the risk ratios for developing overall stroke and ischemic stroke were relatively higher within the first year after head and neck cancer diagnosis compared to other years. Thereafter, a decrease in HR was maintained throughout the follow-up period (Figure 4). However, the HR tendency for hemorrhagic stroke remained relatively constant over time. Furthermore, in the subgroup analysis, men with head and neck cancer showed a slightly higher risk ratio for overall and ischemic stroke compared to women (Table 3). However, in the analysis of hemorrhagic stroke events, we did not detect a significant increase in risk according to sex.

### 3.4. Subtype of Head and Neck Cancer

To elucidate the risk of stroke according to the subtypes of head and neck cancer, we divided the cancer group into sinonasal tract, oral cavity, salivary gland, nasopharynx, oropharynx, hypopharynx, and larynx groups. The results of univariate and multivariate Cox regression models showed that the adjusted HR for incident ischemic stroke events was 1.53 (1.27–1.85) in the oral cavity cancer group, whereas other subtype groups showed no significant adjusted HR (Table 4). Interestingly, we also found that the oral cavity cancer group showed an increased risk of the subsequent development of hemorrhagic stroke (adjusted HR = 1.76, CI = 1.13–2.74; Table 5). Additionally, the incidence of ischemic and hemorrhagic stroke in the oral cavity cancer group was 19.90 and 3.45, respectively.

## 4. Discussion

In the present study, patients diagnosed with head and neck cancer had an increased incidence and a significantly higher risk of incident stroke events. Additionally, the head and neck cancer group showed a higher incidence rate and adjusted HR for newly developed ischemic stroke compared to the non-cancer group, whereas we could not find any association between the two groups in terms of the incidence of hemorrhagic stroke. Moreover, we detected that the risk of overall and ischemic stroke was higher within 1 year after cancer diagnosis and then decreased and maintained a constant value throughout the follow-up period. Furthermore, in this study, men with cancer showed a relatively higher adjusted HR than women. However, when we investigated the risk of stroke according to the subtype of head and neck cancer, we found that patients with oral cavity cancer had a significantly increased risk of developing hemorrhagic stroke during follow-up.

A recent study using meta-analysis demonstrated the incidence of stroke as 1.4% during the first year after a newly detected cancer [4]. Specifically, the risk of ischemic stroke was higher than the risk of hemorrhagic stroke, and the risk for ischemic stroke was particularly high in patients with head and neck cancer. Similarly, our findings showed an increased risk ratio of incident stroke events within one year of cancer diagnosis and a higher risk of ischemic than hemorrhagic stroke. It is well-known that arterial thromboembolism increased in the months prior to cancer diagnosis. Thus, we detected that many studies have found that the risk of stroke increases around cancer diagnosis, and this increased risk can persist for up to 10 years after diagnosis. For these reasons, we thought this might explain why the first year after a new cancer diagnosis has the highest risk of stroke, as the risk declines over time. Although several previous studies have revealed an increased risk of ischemic stroke in patients with head and neck cancer who underwent radiotherapy [14,23,24], a recent population-based cohort study showed that the risk of stroke was not increased in patients with head and neck cancer initially treated with radiotherapy [25]. In this study, we did not compare the risk according to the difference in treatment method, but according to the results of a recent study, our analysis without such a comparison can be said to be meaningful. Interestingly, our study also found that oral cavity cancer was associated with an increased risk of both ischemic and hemorrhagic strokes. A previous nationwide cohort study of oral cavity cancer revealed that radiotherapy could increase the risk of ischemic stroke in patients with oral cavity cancer compared with that in matched normal controls [13]. Another study reported that patients with oral cavity cancer who underwent radio or chemotherapy had a higher risk of an ischemic stroke event [11]. However, contrary to our study, there were no studies showing evidence of increased risk of hemorrhagic stroke in oral cavity cancer.

According to previous studies, cancer patients have a higher risk of stroke compared to the general population, and cancer-related stroke is a major factor in lowering the survival rate of cancer patients [6,7,8,9,10,11,12,13,14,15,16,17,18,19,20,21,22,23,24,25,26,27,28]. Some systematic reviews have also described that the relative risk of stroke is higher in cancer survivors than in the cancer-free population over an unspecified follow-up period [29,30]. To date, several major pathophysiological mechanisms underlying the development of stroke in patients with cancer are as follows: direct tumor effects, cancer complications (coagulopathy and infection), and therapeutic interventions (chemotherapy, radiotherapy, hematopoietic stem cell transplantation, and invasive procedures) [26]. Among these, almost all pathological factors contribute to the development of ischemic conditions due to increased thromboembolic conditions, whereas direct tumor effects, hematopoietic stem cell transplantation, and invasive procedures are known to be the related pathology of hemorrhagic stroke events in cancer patients.

However, our findings still have limitations and require careful interpretation. As representative limitations, first, we only included a limited number of variables because we used a sample cohort dataset. This means that certain factors that may affect stroke incidences, such as smoking and alcohol drinking, could not be adjusted. Second, to classify cancer and stroke, we used diagnostic codes; thus, it was impossible to analyze cancer stage and histology and stroke severity. Third, stroke may be underdiagnosed when patients have severe cancer conditions, such as coma and bedridden status. Fourth, the database we used only provides age as a group due to the de-identification issue. Thus, we matched the two groups by categorizing age data, not their actual age distribution. This means that our study analysis may have residual bias. Fifth, due to privacy security reasons, we have not been able to obtain specific information about the duration of the disease (history of onset) and the severity of the cancer condition. Therefore, we did not know whether the duration or severity of cancer could act as a discriminatory factor in the risk of stroke in cancer patients. Sixth, various treatment methods are used for head and neck cancer, but treatment variables for each patient could not be controlled. Therefore, it was not possible to determine the effect of cancer treatment methods and durations, such as chemotherapy and radiotherapy, on the risk of stroke. Finally, because our study design consisted of a retrospective cohort, this study design could not suggest a pathological mechanism for our findings.

Despite these limitations, our study has important clinical implications. First, we conducted a study with representative data of the entire population distribution and also used the propensity score matching method to control for several important confounding variables that could allow the stroke incidence in the two groups to be compared. Although we could not make a definitive conclusion as to whether the associations we found in our study are newly discovered associations between the two groups or are the result of chance findings, our findings from our nationwide population-based dataset can provide important meanings and hints to real clinical practice. Second, because our dataset is a single-ethnic cohort, the influence of important variables related to stroke incidence, such as race, can be minimized. And since it has a relatively long observation period, the risk presented in this study can be considered a meaningful number. Finally, we minimized surveillance bias for stroke risk in patients with head and neck cancer because we adjusted for all possible sociodemographic characteristics.

## 5. Conclusions

Our findings suggest a possible association between head and neck cancer of the increased subsequent development of stroke, specifically ischemic type. Interestingly, among head and neck cancer, oral cavity cancer survivors were not only associated with an increased risk of ischemic stroke but also with hemorrhagic stroke. Therefore, clinicians should consider cancer survivors at high risk of stroke and pay attention to monitoring related symptoms and physical signs so that stroke onset can be diagnosed and treated early.

## Figures and Tables

**Table 1:** Detailed description of variables of the study participants.

Variables	Comparison (*n* = 5248)	Head and Neck Cancer (*n* = 1312)	*p* Value
Sex			1.000
Male	2228 (42.5%)	557 (42.5%)	
Female	3020 (57.5%)	755 (57.5%)	
Ages (years)			1.000
<45	864 (16.5%)	216 (16.5%)	
45–64	2024 (38.6%)	506 (38.6%)	
>64	2360 (45.0%)	590 (45.0%)	
Residence			1.000
Largest metropolitan	712 (13.6%)	178 (13.6%)	
Other metropolitan	808 (15.4%)	202 (15.4%)	
Other cities	3728 (71.0%)	932 (71.0%)	
Household income			1.000
Low (0–30%)	1360 (25.9%)	340 (25.9%)	
Middle (30–70%)	1756 (33.5%)	439 (33.5%)	
High (70–100%)	2132 (40.6%)	533 (40.6%)	
Charlson comorbidity index			1.000
0	3076 (58.6%)	769 (58.6%)	
1	1300 (24.8%)	325 (24.8%)	
≥2	872 (16.6%)	218 (16.6%)	

Comparison of participants without cancer.

**Table 2:** The risk of incident stroke event in patient with head and neck cancer.

Hazard Ratio	Comparison	Head and Neck Cancer
Stroke		
Unadjusted HR (95% CI)	1.00 (ref)	1.43 (1.21–1.68) ***
Adjusted HR (95% CI)	1.00 (ref)	1.45 (1.23–1.71) ***
Hemorrhagic stroke		
Unadjusted HR (95% CI)	1.00 (ref)	1.44 (0.94–2.20)
Adjusted HR (95% CI)	1.00 (ref)	1.45 (0.95–2.23)
Ischemic stroke		
Unadjusted HR (95% CI)	1.00 (ref)	1.42 (1.19–1.68) ***
Adjusted HR (95% CI)	1.00 (ref)	1.44 (1.21–1.71) ***

HR, hazard ratio; CI, confidence interval. *** *p* < 0.001.

**Table 3:** Hazard ratios of specific diseases according to sex between comparison and cancer group.

Sex	Male		Female	
	Comparison	Head and Neck Cancer	Comparison	Head and Neck Cancer
Stroke				
Unadjusted HR (95% CI)	1.00 (ref)	1.49 (1.15–1.92) **	1.00 (ref)	1.38 (1.12–1.72) **
Adjusted HR (95% CI)	1.00 (ref)	1.52 (1.17–1.97) **	1.00 (ref)	1.40 (1.13–1.74) **
Hemorrhagic stroke				
Unadjusted HR (95% CI)	1.00 (ref)	1.59 (0.83–3.06)	1.00 (ref)	1.34 (0.76–2.35)
Adjusted HR (95% CI)	1.00 (ref)	1.58 (0.82–3.05)	1.00 (ref)	1.34 (0.76–2.36)
Ischemic stroke				
Unadjusted HR (95% CI)	1.00 (ref)	1.44 (1.09–1.89) *	1.00 (ref)	1.40 (1.12–1.75) **
Adjusted HR (95% CI)	1.00 (ref)	1.47 (1.11–1.93) **	1.00 (ref)	1.41 (1.13–1.77) **

HR, hazard ratio; CI, confidence interval. * *p* < 0.05, ** *p* < 0.010.

**Table 4:** Incidence and risk of ischemic stroke event according to the subtype of head and neck cancer.

Variables	N	Case	Person Year	Incidence Rate	Unadjusted HR (95% CI)	Adjusted HR (95% CI)
Cancer type						
Comparison	5248	543	44,749.9	12.13	1.00 (ref)	1.00 (ref)
Oral cavity	949	140	7034.0	19.90	1.62 (1.35–1.96) ***	1.53 (1.27–1.85) ***
Salivary gland	45	2	338.8	5.90	0.48 (0.12–1.92)	0.89 (0.22–3.56)
Oropharynx	51	5	400.7	12.48	1.03 (0.43–2.48)	1.14 (0.47–2.76)
Nasopharynx	59	5	399.8	12.51	1.02 (0.42–2.46)	1.42 (0.59–3.44)
Hypopharynx	30	2	218.8	9.14	0.75 (0.19–3.00)	0.84 (0.21–3.38)
Sinonasal tract	45	2	356.8	5.61	0.46 (0.12–1.85)	0.96 (0.24–3.87)
Larynx	133	11	890.8	12.35	1.01 (0.55–1.83)	1.07 (0.58–1.95)

HR, hazard ratio; CI, confidence interval; *** *p* < 0.001.

**Table 5:** Incidence and risk of incident hemorrhagic stroke event according to the subtype of head and neck cancer.

Variables	N	Case	Person Year	Incidence Rate	Unadjusted HR (95% CI)	Adjusted HR (95% CI)
Cancer type						
Comparison	5248	87	46,713.5	1.86	1.00 (ref)	1.00 (ref)
Oral cavity	949	26	7532.5	3.45	1.81 (1.17–2.81) **	1.76 (1.13–2.74) *
Salivary gland	45	0	341.4	-	0.00 (0–Inf)	0.00 (0–Inf)
Oropharynx	51	1	410.2	2.44	1.29 (0.18–9.26)	1.36 (0.19–9.77)
Nasopharynx	59	1	406.4	2.46	1.29 (0.18–9.26)	1.65 (0.23–11.87)
Hypopharynx	30	0	231.5	-	0.00 (0–Inf)	0.00 (0–Inf)
Sinonasal tract	45	0	358.4	-	0.00 (0–Inf)	0.00 (0–Inf)
Larynx	133	0	922.6	-	0.00 (0-Inf)	0.00 (0–Inf)

HR, hazard ratio; CI, confidence interval; inf, infinite; * *p* < 0.05, ** *p* < 0.010.

## Data Availability

The datasets generated and/or analyzed in the current study are not publicly available due to the policy of the Korea National Health Insurance Service but are available from the corresponding author upon reasonable request.
